# Symptoms, nutritional outcomes and quality of life after total gastrectomy with Roux-en-Y reconstruction: results of a cross-sectional study conducted on 80 long-term survivors

**DOI:** 10.1007/s13304-025-02440-6

**Published:** 2025-10-14

**Authors:** Annamaria Agnes, Alberto Biondi, Marina Carannante, Antonia Strippoli, Francesco Belia, Laura Lorenzon, Roberto Pezzuto, Flavio Tirelli, Lorenzo Ferri, Ilaria Neri, Domenico D’Ugo, Roberto Persiani

**Affiliations:** 1 Dipartimento Di Scienze Mediche E Chirurgiche, Fondazione Policlinico Universitario Agostino Gemelli IRCCS, Largo A. Gemelli N. 8, 00168 Rome, Italy; 2 Università Cattolica del Sacro Cuore, Largo F. Vito N.1, 00168 Rome, Italy

**Keywords:** Gastric cancer, Gastrectomy, Patient reported outcome measures, Quality of life, Dumping syndrome

## Abstract

Impaired functional outcomes are common issues after total gastrectomy, although seldom explored at a long distance from surgery. The primary aim of this study was to investigate the long-term postoperative functional and nutritional results after Roux-en-Y total gastrectomy (RYTG). Patients with gastric neoplasms or *CDH1* mutation undergoing RYTG between 2000 and 2021, with a minimum follow-up of 12 months, were interviewed to assess their functional and nutritional status and to measure their quality of life (QoL) with the GIQLI, EORTC-QLQ20 and EORTC-STO22 questionnaires. Statistical analysis was used to present these results and assess variables associated with body weight variation (BWV), dumping syndrome and QoL. Eighty patients were selected out of 265 RYTGs performed. The mean BWV was − 19.4% ± 9.4. The prevalence of dumping syndrome was 27.5%, the prevalence of food intake disturbances and reflux were 33.8% and 46.2%, respectively. The prevalence of anemia was 27.5%. A higher preoperative BMI and a longer follow-up time were associated with BWV. The only predictor of dumping syndrome was a lower age at surgery. Patients with dumping reported significantly lower GIQLI score [median 132 (IQR 123–138) vs 111 (82–123), *p* < 0.001], and a lower GIQLI score was associated with higher preoperative BMI, with the interaction between dumping syndrome and BWV, and with food intake disturbances. RYTG is associated with a long-term decrease in body weight and a high rate of compromised functional outcomes, with a possible significant effect on QoL. To prevent these occurrences, alternative reconstruction methods to RYTG could be considered.

**Supplementary Information:**

The online version contains supplementary material available at 10.1007/s13304-025-02440-6.

## Introduction

Gastric cancer (GC) represents a global healthcare problem, ranking fifth in cancer incidence and fourth in cancer mortality [1].

Total gastrectomy is the mainstay of curative treatment for locally advanced proximal GC as well as most diffuse GCs and other gastric neoplasms. Moreover, total gastrectomy is a definitive strategy to prevent cancerization in patients carrying *CDH1* mutations [1].

Nevertheless, total gastrectomy has a non-neglectable impact on gastrointestinal function, as documented in several Eastern studies, documenting substantial weight loss, a high prevalence of dumping syndrome, other gastrointestinal disturbances, and impaired nutritional outcomes [2–4]. Most studies have focused on short-term functional outcomes, while only a few have conducted a longer-term follow-up. Moreover, the quality of life (QoL) after total gastrectomy has been poorly analyzed.

Throughout the years, the feasibility of alternative reconstruction methods to total gastrectomy has been assessed to minimize adverse functional outcomes, and some reconstruction methods as the jejunal pouch appeared to have clinical, nutritional, and QoL benefits in several RCTs conducted on the Eastern population [5, 6]. However, given the paucity of data on the functional outcomes after total gastrectomy with Roux-en-Y (RY) reconstruction, especially in the Western scenario, it is still difficult to quantify the potential benefit of alternative techniques to RY total gastrectomy (RYTG).

With this study, we assessed a cohort of patients who had undergone total gastrectomy to address the long-term impact of this procedure in terms of functional and nutritional outcomes and quality of life.

## Methods

### Population of the study and study design

This is a cross-sectional study including all patients who underwent RYTG between 2000 and 2021 for gastric cancer, gastrointestinal stromal tumor (GIST), neuroendocrine carcinoma, lymphoma, or CDH1 mutation, in the General Surgery Unit of the Fondazione Policlinico Universitario “A. Gemelli” IRCCS, Rome—Italy. We included only patients with a minimum time from surgery of 12 months. We excluded patients treated with a palliative aim, patients lost to follow-up, and patients who had insufficient or inconclusive data in the medical records.

### Surgical treatment and postoperative surveillance

Total gastrectomy was performed as a prophylactic upfront procedure for patients with CDH1 mutation and as a curative upfront procedure for patients with proximal early gastric cancer or patients with proximal locally advanced GC that refused or were not candidates for neoadjuvant therapy. Instead, total gastrectomy was performed within a perioperative treatment protocol for all patients with proximal, locally advanced (> cT2 and any N, or T > 2 with bulky node-positive disease), potentially resectable GC. The same approach was applied to patients with neuroendocrine carcinoma according to the existing guidelines [7]. Patients with gastrointestinal stromal tumors (GIST) underwent total gastrectomy for proximal locations not amenable to partial gastrectomy. Patients with gastric lymphoma underwent total gastrectomy for proximal lymphoma, symptomatic and not amenable to undergoing systemic therapy or unresponsive to systemic therapy upon indication of the hematologists.

All patients undergoing total gastrectomy in our center had an RY reconstruction with retrocolic esophagojejunostomy and 50 cm between the esophagojejunostomy and the jejunojejunal anastomosis. Prophylactic gastrectomy was performed with a D1 dissection. In patients with gastric cancer and neuroendocrine carcinoma, surgery was performed altogether with D1 +, D2 or D2 plus lymphadenectomy. In patients with GISTs and lymphomas, lymphadenectomy was performed according to the clinical presentation. After surgery, patients were followed up or administered adjuvant chemotherapy or chemoradiotherapy according to the stages as recommended by the guidelines of the primary disease [7, 8].

### Data collection

Data were retrospectively collected from the medical records and included: clinical aspects (age, gender, preoperative height -cm- and weight -kg- and BMI, administration of neoadjuvant therapy), technical aspects (type of lymphadenectomy), pathological variables (pTNM or ypTNM staging), data on postoperative therapy, and survival data (disease-free survival—DFS). We contacted all patients alive 12 months after surgery to assess the status of their oncologic follow-up and to consent them for inclusion in the present study. They were interviewed by phone with regard to clinical symptoms. The QoL questionnaires (see below) were translated from online modules and patients were instructed on how to complete the online forms or guided through form-filling step by step by a medical practitioner if unable to fulfill the form themselves.

### Endpoints of the study and outcome measures

The primary outcome of our study was the assessment of patients' QoL. QoL was measured with validated Italian translations of the GIQLI [10], the EORTC QLQ-30 and the EORTC STO-22 questionnaires [11, 12] (Supplementary Materials). Scoring was performed according to the absolute and percentage scores (for the GIQLI) and to percentage scores of the subdomains (for subdomains of the GIQLI, EORTC QLQ-30 and EORTC STO-22).

Weight loss was reported as the percentage variation of the weight at follow-up compared to preoperative weight.

The incidence of food intake disturbances (dysphagia or regurgitation) and symptomatic reflux was assessed as a binary outcome (Y/N).

**Fig. 1:**
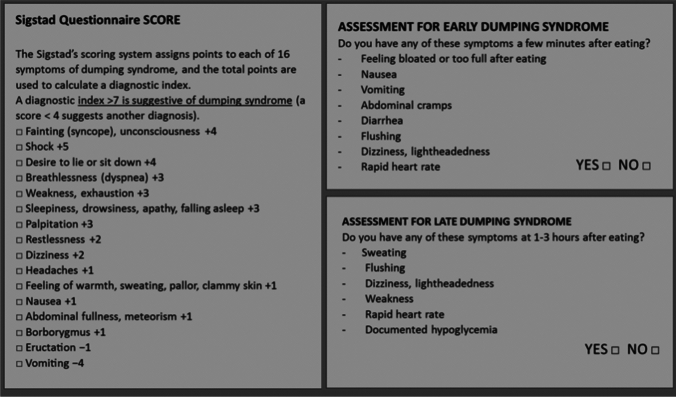
Sigstad Questionnaire and assessment for early/late dumping symptoms

The presence of dumping syndrome was assessed with the Sigstad Questionnaire [9], defining patients with a total score > 7 as those with dumping syndrome. Moreover, symptoms of early or late dumping syndrome were assessed with other two separate sets of questions (Fig. 1).

Nutritional follow-up consisted of an assessment of the prevalence of anemia, of the ongoing iron supplementation, of the vitamin B12 levels at last follow-up, of the ongoing supplementation of vitamin B12, of the vitamin D levels and of the ongoing supplementation of vitamin D. Adherence to nutritional follow-up was also assessed based on this information.

The secondary outcomes were the association between weight loss, quality-of-life scores and the prevalence of dumping syndrome with possible predictors among clinicopathological variables and functional outcomes.

### Statistics

Values were recorded as absolute values and percentages, means and standard deviations, and medians and inter-quartile ranges (IQR), as appropriate, and presented with descriptive statistics. The characteristics of patients with or without dumping syndrome were compared with the Mann–Whitney or t-test for continuous variables, and the Fisher’s exact test or Chi-square test for categorical variables, as appropriate. Correlation analyses were performed to study the association between continuous variables. Linear and logistic multivariable regression analyses were performed to determine the variables associated with the variation in body weight, the absolute GIQLI score after surgery and the presence of dumping syndrome. Initial full models were reduced based on p values for significance (excluding variables with a *p* value > 0.2) and reduced models were tested against full models with the partial F test (for linear models) and the partial likelihood test (for logistic models). A *P* value of < 0.05 was considered to indicate statistical significance. All statistical analyses were performed with Stata 18.0.

## Results

**Fig. 2:**
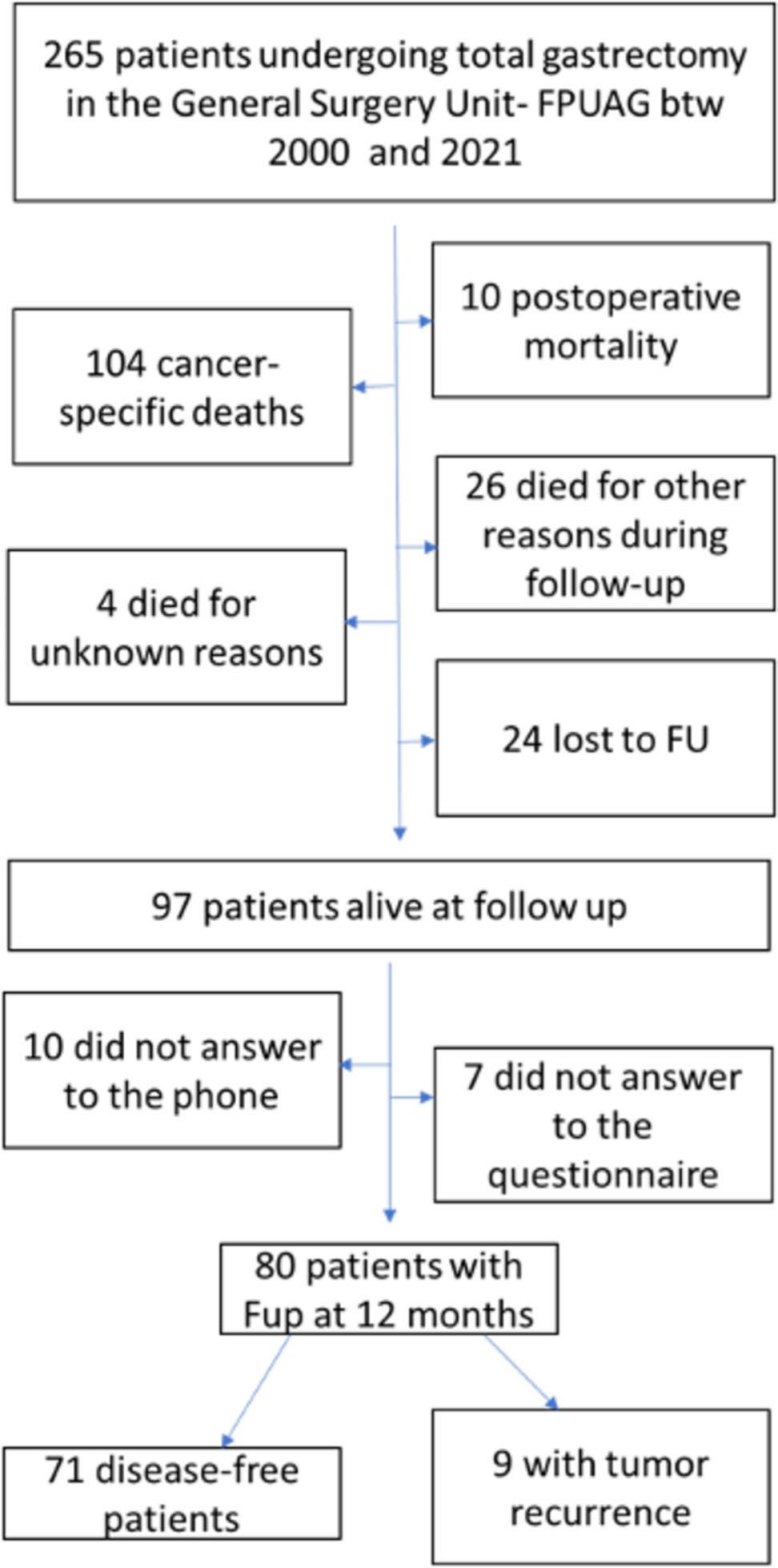
Flowchart of the selection process for determining the sample size of the study

Among 265 patients undergoing total gastrectomy from 2000 to 2021 in the General Surgery Unit of the Fondazione A. Gemelli University Hospital, 80 were selected for inclusion in this study (Fig. 2). The clinicopathological characteristics of these patients are reported in Table 1. The median follow-up of the study was 48 months (IQR 33–92).

**Table 1:** Clinicopathological characteristics and of patients included in the study

	All patients (*n* = 80)	No dumping syndrome (*n* = 58)	Dumping syndrome (*n* = 22)	*P* value
Age at surgery, median (IQR)	67.1 (56–72.9)	70 (60.2–73)	60.1 (46.6–72.1)	0.025
Age at follow-up, median (IQR)	72.7 (63.1–78.8)	75.2 (67.9–79.4)	63.3 (50.6–74.1)	0.003
Gender, n (%)				0.593
- Male	51 (63.75)	38 (65.5)	13 (59.1)	
- Female	29 (36.25)	20 (34.5)	9 (40.9)	
Preoperative weight, mean ± SD	73 ± 13.9	73.5 ± 12.8	71.7 ± 16.7	0.605
Preoperative BMI, median (IQR)	25.6 (23.5–27.6)	25.9 (24.2–27.7)	24.7 (21.8–27.4)	0.100
Primary disease, *n* (%)				0.142
- gastric cancer	76 (95)	56 (96.6)	20 (90.91)	
- GIST	1 (1.25)	1 (1.7)	0 (0)	
- lymphoma	1 (1.25)	1 (1.7)	0 (0)	
- CDH1 mutation	2 (2.5)	0 (0)	2 (9.1)	
Type of lymphadenectomy, *n* (%)				0.667
- D0/D1 lymphadenectomy	7 (8.75%)	6 (10.34)	1 (4.55)	
- D1+/D2 lymphadenectomy	73 (91.25%)	52 (89.66)	21 (95.45)	
Disease stage, *n* (%)				0.475
Stage				
No cancer/complete response/other	6 (7.6%)	4 (6.9)	2 (9.5)	
Stage I	31 (39.2%)	25 (43.1)	6 (28.6)	
Stage II	27 (34.2%)	20 (34.5)	7 (33.3)	
Stage III	15 (19%)	9 (15.5)	6 (28.6)	
Neoadjuvant therapy, *n* (%)				0.580
No	44 (55%)	33 (56.9)	11 (50)	
Yes	36 (45%)	25 (43.1)	11 (50)	
Postoperative complications, *n* (%)				0.091
No	55 (68.75%)	43 (74.14)	12 (54.55)	
Yes	25 (31.25%)	15 (25.86)	10 (45.45	
Adjuvant therapy, *n* (%)				0.495
No	28 (35%)	19 (32.76)	9 (40.91)	
Yes	52 (65%)	39 (67.24)	13 (59.09)	
Presence of cancer recurrence at follow up, *n* (%)				0.700
No	71 (88.75%)	52 (89.7)	19 (86.4)	
Yes	9 (11.25%)	6 (10.3)	3 (13.6)	
Follow-up, median (IQR)	48 (33.4–92.1)	56.8 (33.3–94.2)	46 (33.5–62)	0.346

### Symptoms and nutritional outcomes

The mean weight percentage difference reported at the time of follow-up was − 19.4 + − 9.4%. The prevalence of dumping syndrome was 27.5%, while patients reporting symptoms attributed to early and late dumping syndrome were 27.5% and 28.8%, respectively. The prevalence of food intake disturbances was 33.8% and 46.2% of patients experienced reflux symptoms. Data from nutritional follow-up are reported in Table 2. The prevalence of mild anemia was 27.5%. A vitamin B12 deficit was detected in 3.8% of patients and a vitamin D deficit in 11.3%.

**Table 2:** Functional and nutritional outcomes of patients after total gastrectomy

	All patients (*n* = 80)	No dumping syndrome	Dumping syndrome	*P* value
% Weight variation, mean ± SD	− 19.4 ± 9.4	− 19.9 ± 8.9	− 18.2 ± 10.8	0.468
Dumping (Sigstad), *n* (%)	22 (27.5)	–	–	–
Early dumping symptoms, *n* (%)	25 (31.2)	–	–	–
Late dumping symptoms, *n* (%)	23 (28.8)	–	–	–
Food intake disturbances, *n* (%)	27 (33.8)	15 (25.9)	12 (54.6)	0.015
Reflux, *n* (%)	37 (46.2)	21 (36.2)	16 (72.7)	0.003
Anemia, *n* (%)				0.072
Yes	22 (27.5)	15 (25.9)	7 (31.82)	
Yes, requiring transfusions	2 (2.5)	0 (0)	2 (9.1)	
Iron supplementation, *n* (%)	14 (17.5)	11 (19)	3 (13.6)	0.747
Vitamin B12 levels, *n* (%)				1.000*
Normal	76 (95)	56 (96.5)	20 (90.9)	
Decreased	3 (3.8)	2 (3.5)	1 (4.5)	
Unknown	1 (1.2)	0 (0)	1 (4.5)	
Vitamin B12 suppl, *n* (%)				0.243
No/Occasional	40 (50)	29 (50)	9 (40.9)	
Yes, enteral	20 (25)	13 (22.4)	9 (40.9)	
Yes, IM	20 (25)	16 (27.6)	4 (18.2)	
Vitamin D levels, *n* (%)				0.003*
Normal	69 (86.2)	54 (93.1)	14 (63.6)	
Decreased	9 (11.3)	3 (5.2)	7 (31.8)	
Not assessed	2 (2.5)	1 (1.7)	1 (4.6)	
Vitamin D suppl, *n* (%)				0.137
Yes	33 (41.2)	21 (36.21)	12 (54.55)	

*Calculated excluding missing variables

Patients with dumping syndrome had a significantly higher rate of food intake disturbances (*p* = 0.015), reflux (*p* = 0.003) and vitamin D deficiency (*p* = 0.003).

### Variation in body weight

**Fig. 3:**
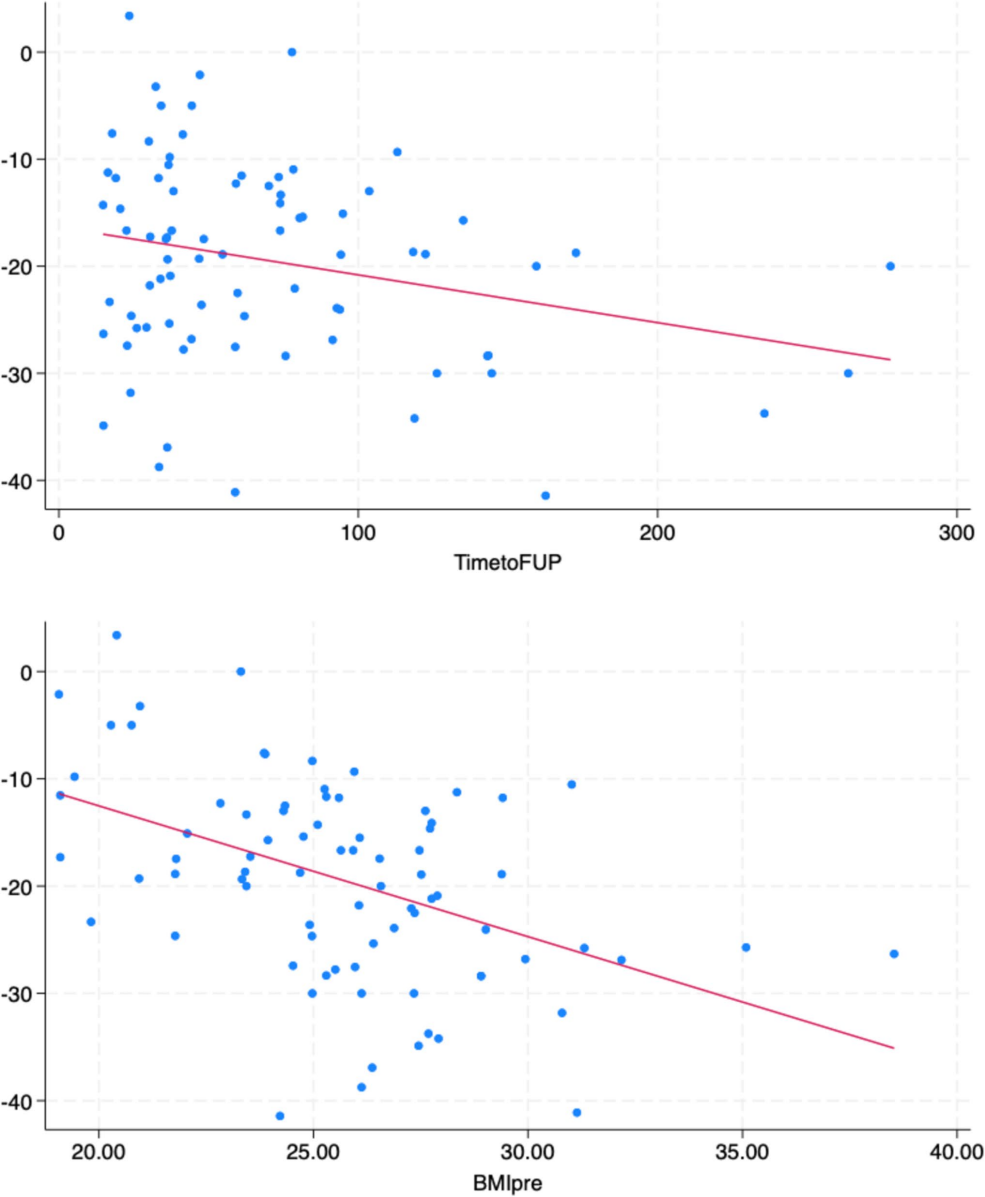
Percentage variation in BW according to the follow-up time (R coefficient −0.1890, Spearman *p* = 0.093) and preoperative BMI (R coefficient −0.468, Spearman *p* < 0.001)

The linear multivariable analysis for variables associated with the variation in body weight (Table 3) identified time from surgery (Coefficient −0.04, CI 95% 0.009–0.075, *p* = 0.014) and preoperative BMI (Coefficient − 1.24, 0.74–1.75, *p* < 0.001) as significant variables. Female gender had a borderline association (Coefficient − 3.72, CI95% − 7.49–0.038, *p* = 0.052) with the variation in body weight. The correlations between the variation in body weight and the time from surgery and preoperative BMI had an R coefficient of − 0.1890 (Spearman *p* = 0.093) and − 0.468 (Spearman *p* < 0.001), respectively (Fig. 3).

**Table 3:** Linear regression for variables associated with the variation in body weight (n = 80 patients)

Variables	beta	*p*	CI95%lower	CI95%upper
Time to follow-up	− 04148	0.014	−0.0744425	−0.0085174
Preoperative BMI	− 1.244437	0.000	− 1.752279	−0.7365953
Gender	− 3.723894	0.052	− 7.486198	0.0384111
Neoadjuvant therapy administration	− 3.435707	0.097	− 7.510784	0.6393708
Adjuvant therapy administration	3.836562	0.068	−.2927343	7.965859
Constant	15.79636	0.024	2.165317	29.4274

Prob > F = 0.0000, R squared of the model = 0.34, adjusted R squared 0.30

### Postoperative quality of life metrics

The postoperative GIQLI median score collected at > 12 months was 127.5 (IQR 114–137). Comparison of these QoL values with those reported in the literature after other common GI surgeries is presented in Table 4. Mean and median values for the GIQLI and subdomains of the EORTC QLQ-30 and STO-22 are presented in Table 1s.

**Table 4:** Mean postoperative values of the GIQLI after different types of GI surgery

Total gastrectomy (this study)	Diverticulitis [13]	Cholecystectomy [14]	Laparoscopic fundoplication [15]	Control cohort [14]	CRS and HIPEC [16]	Bariatrics [17]
Median (IQR) = 127.5 (I114-137) and mean ± sd = 120.1 ± 22.8 at ≥ 15 months postoperatively	Mean ± sd = 118.2 ± 21.0 at 5 years postoperatively	Mean ± sd = 111.71 ± 14.42 at 6 weeks postoperatively	Median (IQR) = 124 (107–128) at ≥ 4 years postoperatively	Mean ± sd = 125.8 ± 13.0	Mean = 100.1, IQR = 89–116 at 12 months postoperatively	Mean ± sd 125 ± 13.1 at 36 months postoperatively

**Fig. 4:**
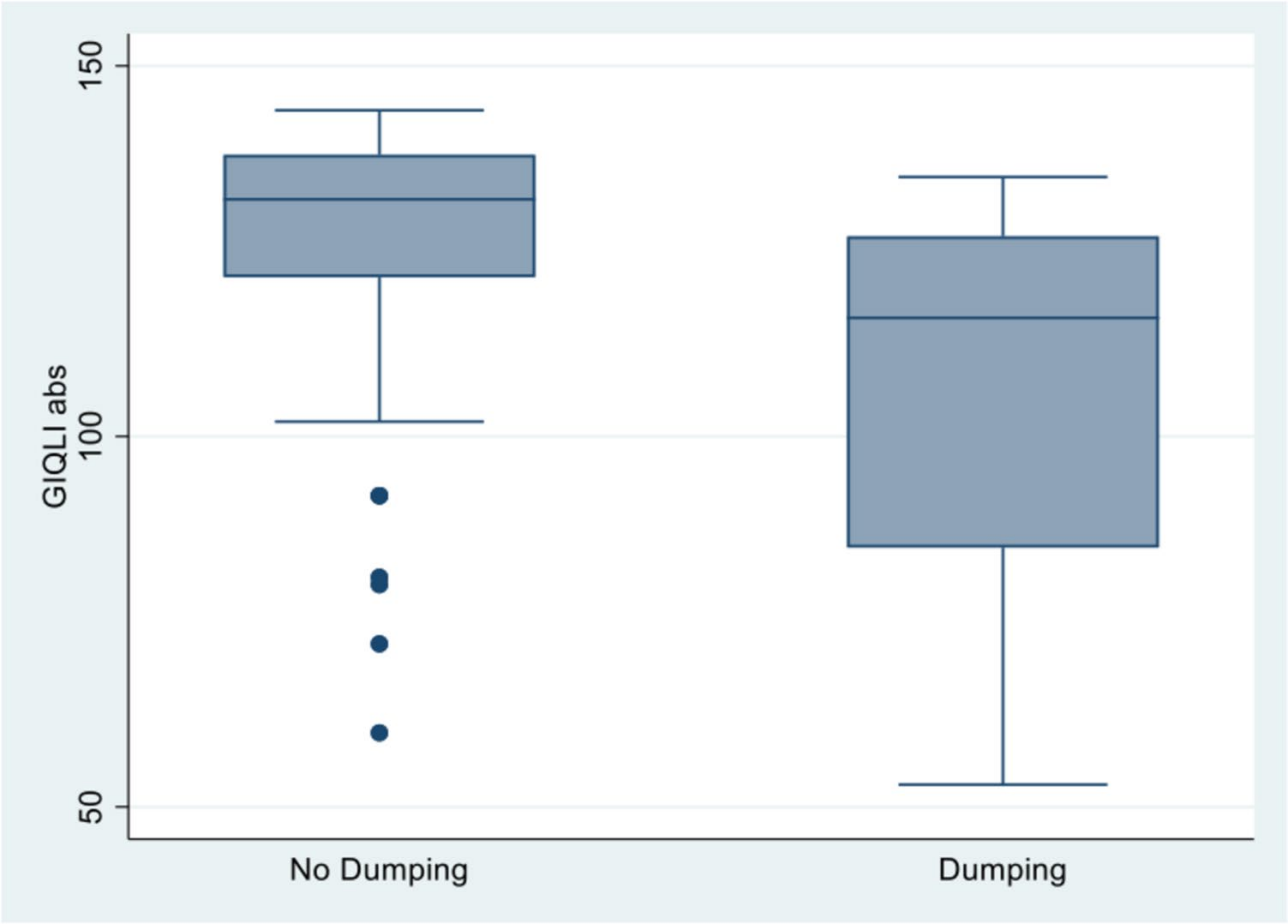
Box plots for the absolute value of the GIQLI score in 71 disease-free patients with or without dumping syndrome [132 (121.5–138 vs 116 (85–127), Mann–Whitney *p* < 0.001)]

**Fig. 5:**
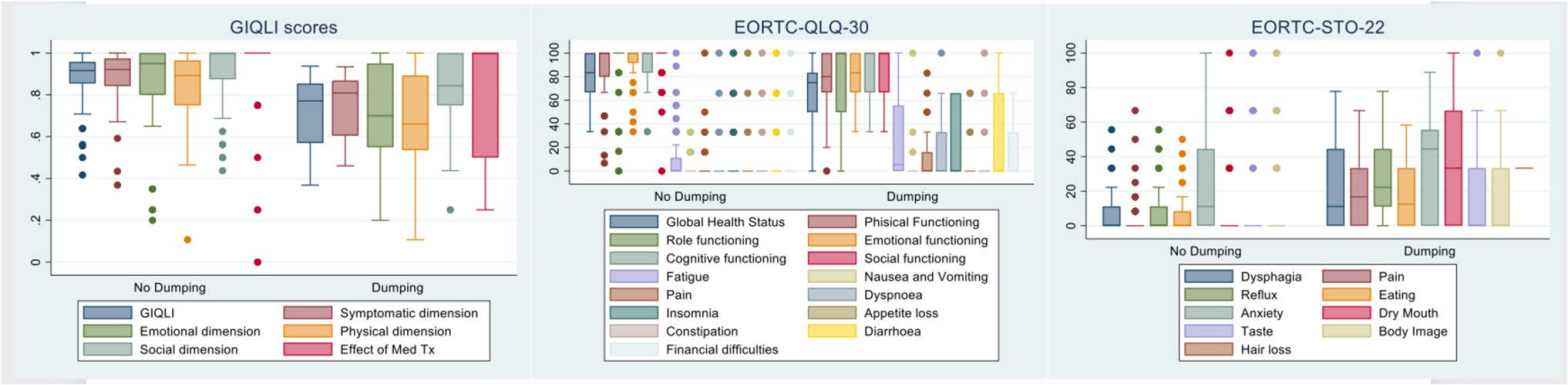
Box plots for the percentage values of the domains of the GIQLI, EORTC QLQ-30 and EORTC-STO22 in patients with or without dumping syndrome

Absolute values for the GIQLI score were significantly different in patients without or with dumping syndrome [132 (123–138) vs 111 (82–123), Mann–Whitney *p* < 0.001], even in the subgroup of patients without cancer recurrence [132 (121.5–138 vs 116 (85–127), Mann–Whitney *p* < 0.001) (Fig. 4). Box plots for the percentage values of the domains of the GIQLI, EORTC QLQ-30 and EORTC-STO22 in patients with or without dumping syndrome are presented in Fig. 5.

The multivariable linear regression for predictors of the absolute GIQLI score (Table 5) identified an association between a higher GIQLI score and a greater preoperative BMI (Coefficient 2.19, CI95% 0.86–3.52, *p* = 0.002), a positive variation in body weight (Coefficient 0.63, CI 95% 0.13–1.13, *p* = 0.015), the presence of dumping syndrome (coefficient −15.88, CI 95% − 26.06–−  5.70, *p* = 0.003) and the presence of food intake disturbances (Coefficient − 12.75, CI 95% − 21.86– − 3.64, *p* = 0.007).

**Table 5:** Linear regression for variables associated with the GIQLI total score, including Interaction term: Dumping##Variation in Body Weight

Variables	Coefficient	*p*	CI95%lower	CI95%upper
Age at surgery	0.3785503	0.065	−0.0236652	0.7807658
Preoperative BMI	2.276363	0.001	.9867451	3.565982
Variation in body weight	0.215944	0.465	−0.3698471	0.8017351
Dumping	9.534922	0.405	− 13.16775	32.2376
Dumping## Variation in body weight (interaction)	1.231956	0.016	0.240434	2.223479
Food intake disturbances	− 14.43264	0.002	−23.34583	− 5.519462
Constant	49.77906	0.018	8.827122	90.73099

F (6,73) = 9.05, Prob > F < 0.001, R-squared 0.43, adjusted R-squared 0.38, partial F test p for change in R2 compared to the regression in Supplementary Table 2: 0.0156

A subsequent linear regression for predictors of the absolute GIQLI score explored the interaction between the presence of dumping syndrome and the variation in body weight, identifying a significant interaction between these two variables (Supplementary Table 2, Supplementary Fig. 1 and 2), consisting of an “activation” effect from dumping syndrome on the influence of the variation in body weight on the GIQLI score: patients without dumping syndrome had no significant effect of the variation in body weight on the GIQLI score, while in patients with dumping syndrome the effect of the variation in body weight on the GIQLI score was significant.

### Dumping syndrome

The multivariable logistic regression for predictors of dumping syndrome identified a lower age at surgery as the only predictor of dumping syndrome in the cohort (Coefficient − 0.074, CI95% − 0.12–− 0.03, *p* = 0.002) (Table 6).

**Table 6:** Logistic regression for variables associated with dumping syndrome

Variables	Coefficient	*p*	CI95%lower	CI95%upper
Age at Surgery	−0.0739556	0.002	−0.1216147	−.0262964
Time to follow up	−0.010989	0.093	−0.0238287	0.0018506
Adjuvant therapy administration	−0.6680449	0.251	−1.809826	0.473736
Constant	4.835277	0.006	1.405066	8.265488

P > Chi2 = 0.004, PseudoR-squared = 0.14, p value (Hosmer–Lemeshow) = 0.5066, area under the ROC curve: 0.7422

## Discussion

In this study, the functional outcomes associated with total gastrectomy were a substantial decrease in body weight and a high prevalence of dumping syndrome, food intake disturbances and reflux even at a long distance from surgery. We observed a relatively high prevalence of mild anemia, while the levels of vitamin B12 and vitamin D were overall adequately controlled, except in the subgroup of patients with dumping syndrome, where vitamin D levels were significantly decreased. Patients with dumping syndrome also had a higher rate of food intake disturbances and reflux. The variation in body weight correlated significantly with preoperative BMI and time from surgery. A lower GIQLI score was associated with lower preoperative BMI, the interaction between dumping syndrome and lower body weight, and food intake disturbances. No identifiable preoperative factor, except a younger age at the time of surgery, was associated with the prevalence of dumping syndrome.

Previous studies of patients undergoing prophylactic gastrectomy had reported similar outcomes in terms of postoperative weight loss after total gastrectomy, ranging within 15–19% of the preoperative body weight [18]. Interestingly, while weight loss has been previously reported to stabilize at about 12 months after surgery [19], in our study, it was correlated with time to follow up, indicating a possible greater weight loss during the years after surgery. This result, although theoretically controlled for by the multivariable analysis, could possibly be influenced by an older age at the time of patient interview, as older patients could have a trend towards a reduction in body weight [20].

Postoperative symptoms have been reported as high as 40% after esophagogastric resections. Among these, meal-related distress, dumping, abdominal pain, and esophageal reflux reported by patients undergoing total gastrectomy were those significantly affecting postoperative QoL [21, 22]. Postoperative symptoms have also been associated with an increase in postoperative depression and anxiety in patients undergoing esophagogastric resection [22]. Our results are in line and validate those of previous studies implicating that the main causes of worse QoL after total gastrectomy are weight loss and the prevalence of symptoms associated with food intake, as dumping syndrome, dysphagia, and reflux. Dumping syndrome also seems to have a key role in determining the perceived impact of the variation in body weight on QoL among patients.

Dumping syndrome has been a well-known complication of gastric resection for several decades [23]. Its physiopathology is still not completely understood, even though it has been partially imputed to rapid emptying of liquids into the jejunum leading to an intravascular fluid shift to the small intestine, as well as to the release of gastrointestinal peptide hormones, including an excessive amount of insulin, which might lead to reactive hypoglycemia. Accordingly, symptoms of early dumping syndrome are mainly gastrointestinal and vasomotor, while late dumping symptoms are associated to hypoglycemia. Challenges in obtaining a univocal definition and diagnostic strategy for dumping syndrome have been reported as well [24, 25]. Total gastrectomy has been associated with a higher prevalence of dumping syndrome and lower QoL after surgery compared to other resections [12, 26]. Several studies have identified predictors of dumping after gastrectomy, associating weight loss, a younger age and total gastrectomy with early dumping syndrome, and weight loss, female gender, BI and total gastrectomy with late dumping syndrome [3]. Another study focused on bariatric surgery has associated dumping syndrome with gender, education level, monthly income, eating more than one large meal per day, and drinking liquids with meals, shifting the focus on life habits and therefore stressing how the treatment should prioritize education on the type of diet and the modality of dietary intake [27]. In this study, the results of the multivariable analysis identified no preoperative predictors of dumping syndrome except a younger age at the time of surgery.

Total gastrectomy is known to face patients with significant changes in their status and life habits, being associated with excessive weight loss, nutrient deficiencies, and metabolic bone disorders. It requires dietary and lifestyle modifications as well as the need for adequate nutritional follow-up [28]. Previous studies have reported how an intensive nutritional follow-up can optimize nutritional outcomes, even though some patients could still suffer from refractory deficiencies due to chronic malabsorption [29]. To promote adherence in these patients, the regional distribution of dedicated nutrition specialists might be preferable compared to excessive centralization, which could lead to the need to overcome a long distance to obtain a proper assessment [30]. In this study, the nutritional follow-up was adequate, and only a minority of patients appeared to be non-adherent to follow-up. While B12 could be safely supplemented enterally or intramuscularly with the great majority of patients having normal vitamin levels, the prevalence of anemia appeared to be higher with a few patients needing transfusions for support. Vitamin D levels also appeared to be controlled in the majority of the cohort (except in patients with dumping syndrome who had a higher rate of vitamin D deficiency), but we did not assess the prevalence of osteopenia or osteoporosis in this population even though they have been reported as emerging problems, especially in patients receiving total gastrectomy at a younger age [31].

To prevent the poorer outcomes reported by patients undergoing total gastrectomy one strategy could be considering changing the type of resection and/or the reconstruction method after total gastrectomy. Proximal gastrectomy has documented functional benefits when compared to total gastrectomy [32] but is currently not recommended outside treatment guidelines for early gastric cancer and is not an option for carriers of a *CDH1* genetic mutation, that require total gastrectomy instead. Alternative reconstruction methods after total gastrectomy are jejunal pouches or functional jejunal interposition. A comprehensive meta-analysis published in 2019 by Syn et al. examined the impact of jejunal pouch reconstruction following total gastrectomy for gastric cancer on surgical, nutritional, and quality-of-life outcomes in 25 studies (17 RCTs and 8 observational studies) involving 1,621 patients. Although pouch reconstruction was associated with longer operative times, it did not increase postoperative complication rates or hospital stay compared to standard Roux-en-Y esophagojejunostomy (RYTG). The pouch was linked to significantly lower rates of reflux esophagitis and dumping syndrome both at 3–6 months and at 12–24 months postoperatively (reflux symptoms: 2.9% vs. 11.7%; dumping syndrome: 2.8% vs. 23.6% for pouch vs. standard reconstruction, respectively). Pouch reconstruction also resulted in better global quality of life scores, improved food intake tolerance, and higher serum albumin levels, with no significant differences in iron or hemoglobin levels between groups [5]. Notably, most of the available evidence derives from patients with early-stage disease, while the safety and complication profile of pouch reconstruction in the setting of locally advanced gastric cancer remains insufficiently defined. Nevertheless, the technique has been applied in several Western centers and from 2021 is recommended by the French Association of Surgery as the technique of choice for reconstruction after total gastrectomy [33]. However, in most European centers, this technique is still not applied due to concerns in terms of complications as well as possible long-term pouch dysfunctions, that have been occasionally reported [34]. These findings underscore the importance of future comparative studies evaluating different reconstruction techniques, particularly in terms of their impact on quality of life and nutritional outcomes.

In this perspective, our results are a valuable reference to adequately counsel patients and to target future interventional studies. This is one of the first studies systematically addressing long-term patient-reported outcomes after total gastrectomy in the Western population. Its limitations are imputable to the monocentric nature and retrospective design, to the sole collection of postoperative outcomes and to the heterogeneity of the timing of data collection, that were, however, controlled for in the analysis. These elements could limit the generalizability of our findings, which would be strengthened by future multicentric and/or prospective studies to validate our results in larger and more diverse cohorts. For this study, we employed the GIQLI and EORTC questionnaires, which are widely available and validated in Italian. There are also two newly developed integrated questionnaires, the Japanese Post-Gastrectomy Syndrome Assessment Scale (PGSAS)−45 and the Korean KOQUSS-40, that have a specific focus on post-gastrectomy syndrome [25, 35], which were not employed in this study as they are currently not validated in Italian.

A last consideration is that our study did not focus on short-term functional outcomes after surgery. While our results could be useful to optimize outcomes for long-term survivors, assessing the implications of poor functional outcomes in the short-term timeframe could give insight into the influence of gastrointestinal issues on the time to return to chemotherapy and on the tolerance to adjuvant treatment.. Moreover, it would inform on the time needed to reach a stable weight and outcomes after gastrectomy.

## Conclusions

Total gastrectomy is associated with a consistent decrease in body weight and a high rate of compromised functional outcomes even in the long-term. Maximal effort should be employed to identify patients at risk of compromised outcomes and to develop effective treatments to minimize their impact on QoL. To further prevent these occurrences, the exploration of alternative reconstruction methods to Roux-en-Y total gastrectomy could be considered.

## Supplementary Information

Below is the link to the electronic supplementary material.

Supplementary file1 (DOCX 140 KB)

## Data Availability

Datasets analyzed during the current study are publicly available upon reasonable request.
