# Supplementary material for: Symptoms, nutritional outcomes and quality of life after total gastrectomy with Roux-en-Y reconstruction: results of a cross-sectional study conducted on 80 long-term survivors
Source: Updates Surg. 2025 Oct 14;78(3):1047–58. doi: 10.1007/s13304-025-02440-6 (PMC13249924; doi:10.1007/s13304-025-02440-6)
Supplement: Supplementary file 1 — Supplementary file1 (DOCX 140 KB) [file 13304_2025_2440_MOESM1_ESM.docx]

**Title**

Symptoms, nutritional outcomes and Quality of Life after total gastrectomy with Roux-en-Y reconstruction: results of a cross-sectional study conducted on 80 long-term survivors

**Authors**

Annamaria Agnes^1^, Alberto Biondi^1,2^, Marina Carannante2, Francesco Belia^2^, Laura Lorenzon^1^, Roberto Pezzuto^1^, Flavio Tirelli^1,2^, Lorenzo Ferri^1^, Ilaria Neri^2^, Domenico D’Ugo^1,2^, Roberto Persiani^1,2^

**Affiliations**

1 Fondazione Policlinico Universitario Agostino Gemelli IRCCS, Largo A. Gemelli n. 8, 00168, Rome, Italy

2 Università Cattolica del Sacro Cuore, Largo F. Vito n.1, 00168, Rome, Italy

**Supplementary Materials - Index**

| **Type - Supplementary Figures and Tables** |  |
| --- | --- |
| 2 Supplementary Tables  2 Supplementary Figures |  |

**Supplementary materials**

| Supplementary Table 1 – Quality of life measured with the GIQLI, EORTC QLQ-30 and EORTC STO22 | | |
| --- | --- | --- |
|  | Mean (SD) | Median (IQR) |
| **GIQLI Absolute score:**  **GIQLI absolute and domain scores (%), mean (SD):**  Absolute score  Symptomatic dimension  Emotional dimension  Physical dimension  Social dimension  Effect of Medical Treatment | 120.1 (22.8)  83.4 (15.8)  84.7 (15)  81.3(23)  78.2(22)  88 (17.6)  87.8 (26.1) | 127.5 (23)  88.5 (15.9)  89.5 (18)  90 (30)  85.7(23.3)  100 (18.7)  100 (0) |
| **EORTC QLQ-30 domain scores (%):**  Global Health Status  Phisical Functioning  Role functioning  Emotional functioning  Cognitive functioning  Social functioning  Fatigue  Nausea and Vomiting  Pain  Dyspnoea  Insomnia  Appetite loss  Constipation  Diarrhoea  Financial difficulties | 78.4 (21.5)  84.6 (23.6)  86.3 (29)  88.6 (17.9)  91.3 (15.9)  89.6 (25.5)  15.8 (26.4)  4.3 (13.4)  7.4 (19.2)  9.5 (22.5)  14.5 (28.3)  5.8 (18.1)  6.6 (20.7)  14.5 (26.8)  9.5 (22.4) | 83.3 (33.3)  100 (26.7)  100 (0)  100 (16.7)  100 (16.7)  100 (12.5)  0 (22.2)  0 (0)  0 (0)  0 (0)  0 (0)  0 (0)  0 (0)  0 (33)  0 (0) |
| **EORTC-STO22 domain scores (%):**  Dysphagia  Pain  Reflux  Eating  Anxiety  Dry Mouth  Taste  Body Image  Hair loss | 12.1 (18.5)  7.9 (15.3)  12.8 (18.1)  9.8 (14.8)  25.3 (28.8)  21.3 (33.2)  10.4 (25.8)  13.3 (27.4)  0.4 (3.7) | 0 (22.2)  0 (8.33)  0 (22.2)  0 (16.7)  11.1 (44.4)  0 (33.3)  0 (0)  0 (0)  0 (0) |

| Supplementary Table 2 – Linear regression for variables associated with the GIQLI total score | | | | |
| --- | --- | --- | --- | --- |
| Variables | Coefficient | p | CI95%lower | CI95%upper |
| Age at Surgery | .1801513 | 0.350 | -.2015094 | .5618119 |
| Preoperative BMI | 2.187799 | 0.002 | .8565537 | 3.519044 |
| Variation in Body Weight | .626454 | 0.015 | .1263488 | 1.126559 |
| Dumping | -15.88059 | 0.003 | -26.06248 | -5.6987 |
| Food Intake Disturbances | -12.74895 | 0.007 | -21.8568 | -3.6411 |
| Constant | 73.11376 | 0.000 | 35.48996 | 110.7376 |

Supplementary Figure 1: Scatter plot illustrating the distribution of the GIQLI score (y) based on the variation in body weight (x) and the presence of dumping syndrome (no dumping= blue dots, dumping= red dots) according to the interaction detected in the multivariable regression from Table 5

Supplementary Figure 2: Prediction of the GIQLI score (y) based on the variation in body weight (x) and the presence of dumping syndrome according to the model presented in Supplementary Table 2.

| Supplementary Table 2 – Linear regression for variables associated with the GIQLI total score | | | | |
| --- | --- | --- | --- | --- |
| Variables | Coefficient | p | CI95%lower | CI95%upper |
| Age at Surgery | .1801513 | 0.350 | -.2015094 | .5618119 |
| Preoperative BMI | 2.187799 | 0.002 | .8565537 | 3.519044 |
| Variation in Body Weight | .626454 | 0.015 | .1263488 | 1.126559 |
| Dumping | -15.88059 | 0.003 | -26.06248 | -5.6987 |
| Food Intake Disturbances | -12.74895 | 0.007 | -21.8568 | -3.6411 |
| Constant | 73.11376 | 0.000 | 35.48996 | 110.7376 |

Prob>F <0.001, R-squared 0.38, adjusted R-squared 0.34
